# The Integration of Wheelchair Users in Team Handball

**DOI:** 10.3390/sports9120168

**Published:** 2021-12-14

**Authors:** Steffen Greve, Sinikka Heisler, Pia von Keutz, Blall Shirdel, Frowin Fasold

**Affiliations:** 1 Institut of Physical Activity, Sports and Health, Leuphana University of Lüneburg, 21335 Lüneburg, Germany; 2 Department of Performance Psychology, German Sport University Cologne, 50933 Köln, Germany; sinikkaheisler@web.de; 3 Institute of Exercise Training and Sport Informatics, German Sport University Cologne, 50933 Köln, Germany; pia.flummi@gmx.de (P.v.K.); F.Fasold@dshs-koeln.de (F.F.); 4 Faculty of Education, Universität Hamburg, 20148 Hamburg, Germany; balu22309@hotmail.com

**Keywords:** wheelchair handball, organized sports, inclusion, contact theory, multi-method design

## Abstract

Thus far, there are only a few sports activities in which people with and without intellectual disabilities can participate together and on an equal footing. The situation is even more complicated when people who are dependent on a wheelchair want to take part. The sports project Freiwurf Hamburg aims to make team handball playable for everyone. This case study documents how this can be achieved with a modified version of the handball game for runners and wheelchair users. Qualitative and quantitative data are collected and evaluated. The results show that players tend to distinguish between the roles of runner and wheelchair user rather than between disabled and non-disabled.

## 1. Introduction

In organized sports in Germany, people with and without disabilities rarely participate together [1]. After the United Nations (UN) ratified the Convention on the Rights of People with Disabilities, many sports organizations have committed themselves to foster inclusive sports opportunities [2]. Freiwurf Hamburg is one of these local initiatives that allows people with and without disabilities to play together in an inclusive team handball league. Freiwurf has approx. 100 players, about 75 of whom have an intellectual disability and the others are without disabilities. However, up to now, persons using wheelchairs are not able to participate in the matches in competition. This is in conflict with the initiative’s goal of “handball for everyone” [3]. As a result, Freiwurf aims to create possibilities for people with and without wheelchairs to play team handball together. It is already a big task to carry out competitive sport events for people with and without disabilities. If we now include different disabilities, and in addition players in wheelchairs, the task becomes even more difficult. The present case study exemplarily shows one way of designing an integrative sports competition. Additionally, previous studies revealed group hierarchies within the teams of Freiwurf that are based on a mental categorization into players with and without disabilities [4,5]. Similar results are also available for Special Olympics Unified Sports [6,7]. Freiwurf wants to dissolve these hierarchies. The following case study was developed as an attempt to realize the inclusive goal of an inclusive sports initiative (Freiwurf Hamburg) by applying a mixed-method design.

## 2. Inclusion in a Sports Club

Following the UN Convention, organized sports were given the task of enabling people with disabilities to participate in their offerings [2], which have since been tailored to the needs of this target group on the grounds of their right to participation. Organized sports have the task of facilitating and stimulating this exchange at club level [8].

One example is the handball initiative Freiwurf, an association of eight teams from five clubs, which has been awarded as best practice for inclusive sport by the National Paralympic Committee Germany [8]. The participating teams consist of male and female players with and without intellectual disabilities and play according to the current rules of the German Handball Federation within a league system. A canon of values defines the framework of the inclusive competition format. The goals listed there are, for example, “fun at the game” and the prioritizing weighting of the “stake of our players” [3] before result orientation. However, studies on Freiwurf have shown that, for various participants with disabilities and coaches, sporting success in the sense of winning matches and championships is also quite important [4,9]. However, the role of players without disability also appears to be important, some of whom were described as “helpers for the disabled” [4] in the context of the studies mentioned above. Here, the focus seems to be on a kind of social service rather than on sporting activity (e.g., for Unified Sports: [6]). However, previous attempts at creating an inclusive sports setting have not considered adaptations for wheelchair users. This is necessary in order to meet the inclusive goal of sports initiatives, as currently, a group of people are excluded from competing in sports. In this way, the case study also allows for analysing the social interactions, hierarchies, and general sports enjoyment of different groups of participants in an inclusive sports format.

In this study, the definition of inclusion in sports is to be understood as the most comprehensive possible description of the term for solving the problem being addressed. For Kiuppis [10], the recognition of people with disabilities as athletes in sporting competitions is important. In this way, inclusion in organized sports can be interpreted as encompassing appropriate cultures (an appreciation and affirmation of heterogeneity), structures (of all people regardless of prior experiences in the sports club), and practices (in the planning and execution of training and competition) [8]. Following the bio-psycho-social model of disability [11], the present study is based on this conceptual interpretation. The individual thus moves to the centre, and the contextual conditions that disadvantage people with disabilities are focused on.

There is little research in the field of inclusive club sports in Germany [12]. Internationally, however, some research results can be found. The results of studies of the Special Olympics Unified program seem interesting for this investigation, as people with and without intellectual disabilities play sports together. However, these studies document the relationship between unified athletes and partners [13,14]. No prior studies have been conducted on targeted interventions concerning the testing of play forms in which wheelchair users and runners, and those with or without intellectual disabilities, play together.

## 3. Contact Theory

Direct contact between people with and without disabilities is assumed to be a necessary condition to evoke a positive attitude towards the other group [15]. Effects may include correcting misconceptions about people with disabilities, divesting them of otherness, and establishing trust in this group [16]. In general, Allport’s contact theory [17] offers an approach to describing and analysing the phenomena occurring in Freiwurf. He formulates optimal conditions to combat prejudices, which are similarity in group status, pursuance of the same goal by majority and minority groups, support of the contact by public organizations, and the existence of a shared interest by all group members.

The goals of Freiwurf seek to realize these conditions [4]. All club members have the same status as handball players and share the main goal of improving their game-related skills. However, previous studies reveal that this goal is not always experienced and realized by all members [18]. This leads to the research question of why direct contact only has a positive effect on certain people or situations in Freiwurf. Pettigrew’s explanation [19] that people with prejudice avoid intergroup contact does not fit this case, because previous studies of Freiwurf show that members without disabilities show a positive attitude towards those players with disabilities [4]. However, Pettigrew [19] underlines that the conditions described by Allport [17] overlap and interact with each other. For Pettigrew, it is important how and why direct contact changes attitudes and behaviours [19]. Furthermore, individual differences can shape, as well as social norms do, the effects of intergroup contact [19]. Based on the findings of their meta-analysis, Pettigrew and Tropp [20] highlight five processes that take place during direct contact: learning about the outgroup, change of behaviour, generating affective relations, reassessment of the ingroup, and reduction of intergroup anxiety. They also point out that positive intergroup experiences are generalized across situations [20].

Previous research at Freiwurf has shown that players without disabilities value new positive qualities of players with disabilities, for example their performance capability [18]. However, negatively affected processes also take place. Players with disabilities who assess their ingroup (players with disabilities) as the wrong players and outgroup (players without disabilities) as the right players remain reserved towards players with disabilities.

Cloerkes [16] specifies three favourable conditions that are directly related to a positive interaction of people with and without disabilities: voluntary contact, intensity of the relationship, and expectance of reinforcement from the social relation. The first condition applies in Freiwurf, as participation is voluntary, and it can be assumed that people who would reject contact do not participate. As for the second condition, the intensity of relationships between the members of Freiwurf is clearly visible to an observer. For the third condition, Cloerkes [16] claims that, because of a perceived low social attractiveness of people with disabilities, relationships between people with and without disabilities can never be balanced. Therefore, people without disabilities usually avoid social interaction with people with disabilities. Thus, reinforcement can arise out of the perceived gratefulness of people with disabilities and out of their own moral consciousness of being a good person [16]. This can be observed in Freiwurf in the case of disability assistants [4].

## 4. Wheelchair Handball and Inclusive Handball

**Figure 1:**
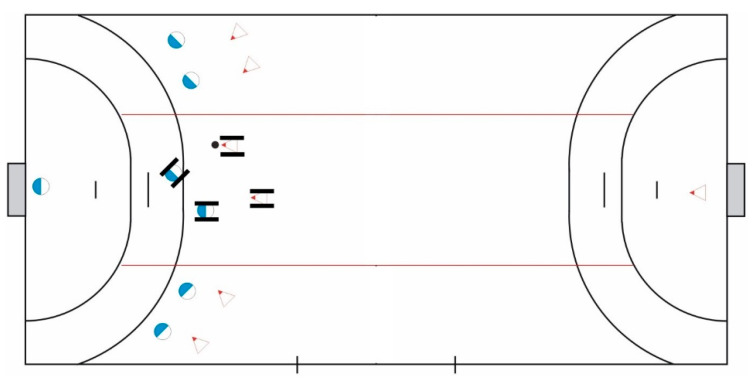
Playing field for inclusive handball: central zone for wheelchair users; two zones on the side for runners; no halfway line.

In a pilot study, two handball game forms, wheelchair handball and inclusive handball, were played in a practice session of Freiwurf [21]. This session was followed by a guided interview [22] and a group discussion with the coaches [23]. In wheelchair handball, all players, both those with and those without disabilities, use a wheelchair. The second game form, presented by Ernenwein [24], represents a modified version of handball and wheelchair handball was given the name “inclusive handball”: The playing field is divided into three elongated zones. Runners and wheelchair users are allowed to leave their zones but are not allowed to intervene in the game outside of their zone (Figure 1) [25]. Additionally, in this form of the game, some players without physical disabilities use a wheelchair to play handball.

The results of the pilot study reveal a positive attitude and sports enjoyment of the participants towards both games and a common experience of disabled and non-disabled athletes. Sports enjoyment plays a major role for the assessment, and according to Birrer and Stirnimann [26], it plays a major role for lifelong sports participation. Moreover, some athletes benefit from the new experience using a wheelchair because it opens up new perspectives and understanding for wheelchair users. A striking observation is that the participants refer to both runners and wheelchair users as “we”, when reporting their experience with a wheelchair. The lack of differentiation between players with and without (intellectual) disabilities shows that hierarchies have become perhaps irrelevant in this moment. This indicates a possible potential for the game approach of inclusive handball by integrating wheelchairs to counter the problem of hierarchy.

## 5. Research Question

Freiwurf [3] states that ingroup differentiation and hierarchies based on disabilities should not play a role in their activities. The contact hypothesis [19] suggests that people with and without disabilities playing team handball together would comply with the goals of Freiwurf. However, in previous studies in the context of Freiwurf, hierarchization could be reconstructed [5,9]. On the other hand, the pilot study indicates that playing wheelchair handball and inclusive handball could have a positive influence on ingroup differentiation and hierarchies based in disabilities.

The study follows the research question of how players with intellectual disabilities interpret and experience the game of “inclusive handball”, which includes zones for athletes playing in a wheelchair and zones for athletes playing without a wheelchair. The perspectives of the players are very interesting, because the players are the ones for whom the “inclusive handball” offer is designed. Another interest of the study is to test the playability of the game “inclusive handball”. This will be studied for the first time by testing the “playing experience” (character of the game) of “inclusive handball”, as well as group thinking and sports enjoyment of the players without intellectual disabilities. These quantitative data serve to gain further knowledge that the reconstruction of the subjective perspectives of players with disabilities may not provide.

## 6. Method

### 6.1. Design

To answer the research question, a qualitative dominated multi-method design is used [27]. The starting point is the view of the participants with disabilities on the game of “inclusive handball”. As described above, this concerns the situation of the players with disability in the teams, and the sports enjoyment is also relevant. Therefore, two quantitative instruments came into focus for the survey with the participants: The Inclusion of the Other in the Self Scale (IOS; [28]) to assess group thinking; and the Magglinger Sport Enjoyment Scales (MSES; [26]) to measure sports enjoyment. For people with an intellectual disability, questionnaires are less suitable [29]. Interview guidelines were, therefore, developed on the basis of these two questionnaires. This was used to interview people with intellectual disabilities. The people without intellectual disabilities filled in the questionnaires. In addition, field notes (e.g., communication between players or between players and coaches) were written during the intervention, and the games were filmed for post hoc game experience analysis. In this way, the case study of an inclusive handball tournament consists of four parts, which are interwoven: Interviews (qualitative approach A), field notes (qualitative approach B), questionnaires (quantitative approach A), and video observations (quantitative approach B). According to Kuckartz [30], the mixing of the data in the discussion of the results is useful and is applied here. The qualitative results from qualitative approach A were compared to the data of qualitative approach B and quantitative approach A and B.

### 6.2. Intervention

A tournament of all participating clubs in Freiwurf was organized. In all, 58 athletes (38 males, 20 females) participated in the tournament. Of them, 48 were people with intellectual disabilities (34 males, 14 females), and 10 were people without intellectual disabilities (4 males, 6 females). The athletes were randomly divided into seven teams. In total, eight matches, each with a playing time of ten minutes, were played. Many players who do not use wheelchairs in everyday life tried out playing handball in wheelchairs. Additionally, the coaches held two group discussions, lasting a total of approximately 10 min.

### 6.3. Data Collection and Material

#### 6.3.1. Qualitative Approach A (Interviews)

Due to the lack of prior research, this study had an explorative character. In order to learn more about the perspectives of the participants, following the tournament, 30 (21 males, 9 females) guided interviews, lasting 8 to 17 min each, were conducted to assess the opinions of the participants with disabilities. All interviewed athletes had intellectual disabilities. One athlete also uses a wheelchair in everyday life. Moreover, 18 participants of the tournament (13 males, 5 females) did not want to give an interview.

Since interviews with people with intellectual disabilities represent a particular challenge [31], appropriate measures were taken. In a stimulated recall procedure, photos of game situations were used during the interview in connection with the questions being asked [32]. Interviewing people with an intellectual disability [33] requires using open questions to avoid possible unconsidered and uncritical agreement from the interview partner. The questions were written in plain language [34]. For example, the questionnaire item “The activity has (not) encouraged mutual support.” was transformed into the interview question “How did you help each other in the tournament?” All interviewers were present at the tournament and in several preceding practice sessions in order to meet the test persons and establish a feeling of rapport. In preparation, the interviewers discussed the importance of taking their interview partners seriously and supporting their autonomy irrespective of their answers.

As understanding subjective individual actions and perspectives is the focus of this study, social actions and processes were analysed and compared, a lack of theoretical processing of the topic was found and work was carried out at the same time every day. The interviews were transcribed and analysed according to the coding strategies of the methodology grounded theory [35]. Only open and axial coding were used. Selective coding and core category elaboration were omitted. The development of an object-anchored theory was not at the centre of interest at this point. It was possible to describe phenomena referred to in responses to the interview questions, so that the coding methods appeared sufficient. The phenomena and categories identified after the first open coding phases were confirmed as relevant and developed by further data collection and axial coding.

#### 6.3.2. Qualitative Approach B (Field Notes)

Field notes were taken during the whole intervention, including all games and all discussions among coaches and officials. A total of 167 field notes on the matches and the coach discussions were taken by four independent observers. Some of the coach’s statements were recorded exactly as written. Structuring qualitative content analysis following Mayring [36] was used to structure and interpret the notes. In this deductive approach, categories were defined in advance. These categories were also based on the parameters of the MSES and IOS scales. The observers were student workers who had attended several Freiwurf training sessions prior to the intervention. For this purpose, the observers were informed in advance about the tournament and the objectives of the case study. It must of course be noted here that the observers’ notes usually contain personal and subjective comments. However, it can be assumed that the observers had professional competence due to the measures taken in advance, so that these comments serve the interest in knowledge.

#### 6.3.3. Quantitative Approach A (Questionnaires)

Immediately after the tournament, the 10 players without disabilities completed questionnaires. Group thinking was evaluated using the IOS scale of Aron et al. [28]. In a Venn diagram, the test persons were asked to indicate the extent to which they perceived themselves as part of a group.

Sports enjoyment was measured using the MSES scale of Birrer and Stirnimann [26] by means of four different factors: perceived skill level, social interaction and support, special movement experiences, and level of enjoyment of the physical activity. Each factor consisted of four items. On a seven-point Likert scale, the test persons were asked to rate their experience on a continuum between two opposite statements (e.g., I had a lot of fun in the activity, and I did not have any fun in the activity). The questionnaire included five additional questions to gather sociodemographic data as well as the level of experience in team handball and in the handling of a wheelchair. For the evaluation, the means of the four different subscales were used, with a high mean representing a high parameter value in the category. For the items of the four categories, Cronbach’s alpha was >0.73. Therefore, the reliability of the scale is assumed to be satisfactory [37].

#### 6.3.4. Quantitative Approach B (Video Observations)

The game experience (game character) is described using data from a post hoc video analysis of the first four matches, which were recorded during the tournament. To generate reference data, Freiwurf provided videos from regular matches.

Three factors were analysed to assess game experience. Game flow is based on Kalyvas and Reid [38] and was determined through a comparison of on time and off time as well as the number of successful passes. On time is defined as the time in a match when the ball is in play, and off time is the time when it is not in play. Passes during off time were not included in the analysis. The level of playful cooperation was assessed by the number of passes (successful and unsuccessful). The involvement in play-related situations was evaluated by the average number of actions with the ball per minute for each player (excluding the goalkeeper). These situations include passing the ball, moving with the ball towards the goal, attempting to score, blocking a shot, intercepting a pass, competing for a free ball. The same parameters were evaluated in four randomly selected 10-min video segments of a regular match of Freiwurf (season 2017–2018).

## 7. Results

**Figure 2:**
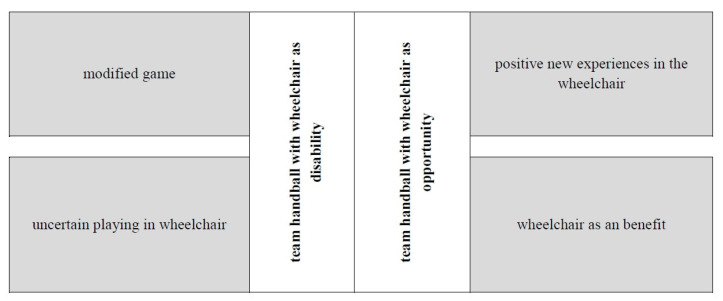
Categories and head categories derived from the interviews with the players with disability.

The results are presented using four categories derived from the interviews with the players with disabilities. This prioritization results from the research question of how the athletes with disabilities interpret and experience the game of inclusive handball. Two general categories were formed. The categories modified game and uncertain playing in wheelchair were assigned to the general category wheelchair as disability, whereas the categories positive new experience in wheelchair and wheelchair as benefit were assigned to the general category wheelchair as opportunity (Figure 2).

The categories are illustrated by exemplary statements. For verification, validation, and new insights, they are combined with the results of the parts qualitative approach B and quantitative approach A and B [27,30,39].

### 7.1. Interpretation of the Categories

#### 7.1.1. Category: Modified Game

Playing inclusive handball results in changes in the idea of the game and demands new physical skills on different levels. Several statements of the players with disabilities illustrate this opinion. The athletes describe a significantly slower paced game, which is less dynamic in comparison to the regular game at Freiwurf. The following quote of Felice (all names were changed) underlines this view (all quotes are translated from German to English without corrections).

“Well, I think the wheelchairs reduce the pace of handball a bit. And a handball game without speed is in my opinion not handball at all, because everything we did in training we couldn’t do here as we wanted, because we still had to show consideration for the wheelchair users.”

(line 14ff)

Felice describes her experience of a slower game in comparison to the game she is used to. In her opinion, the speed of the game determines its quality. She goes even further as she says the name team handball should not be used for the modified game with wheelchairs because of the missing game dynamics. Furthermore, she points out that the usual tactics cannot be applied in consideration of the wheelchair users. Obviously, Felice would rather use the skills she is familiar with rather than develop the new ones that inclusive handball demands. Furthermore, the interviews reveal difficulties in players using certain skills in a wheelchair.

In the table below, we see that the phenomenon of a slower, less dynamic game is confirmed in the analysis of the data on game experience gathered during video game analysis in the quantitative approach B. Significantly fewer successful passes were completed in comparison to the regular matches at Freiwurf.

Additionally, the players mentioned that they were confused by the new rules and tactics. Due to the wheelchair users, all of the playing positions of the regular game, such as pivot and centre back, cannot be filled by runners. For some players, this appears to be an unsolvable challenge, as stated by Anton.

“Uhh, that was … Well I thought it was strange with the wheelchair users, that you must have a boundary. Well, all that running along the side, because I am actually used to always playing as a pivot and I wasn’t allowed to go into the centre and I didn’t understand that, because yes. As I said, a girl once said that I was standing in the wrong place, because I wasn’t able to wait.”

(line 2ff)

Anton would like to use familiar tactics, too. He described his confusion and distress in the situation, which made him impatient and upset him. Problems with the different zones are also described in the field notes:

“Player with disability in a wheelchair does not understand the new boundaries and is permanently in the wrong zone and half [thus often three wheelchairs are in one zone instead of four as required].”

(line 112)

This is an exemplary statement for the code regulations and wheelchairs, in which all observations concerning problems with the rules of inclusive handball and in the handling of the wheelchair are summarized. Similar negative events are also described in the following category.

#### 7.1.2. Category: Uncertain Playing in Wheelchair

Several statements of athletes reveal perceived uncertainties in relation to the new conditions. Fear of injuries emerges as an important aspect, which is also caused by distress due to the unusual challenges. This is underlined by Norbert’s statement.

“When I have a game, but there you certainly noticed it, that also really exhausts you. You have to prepare yourself physically and mentally, mhh I can’t do so much now. I just jump along the whole field, but there you have to assess the dangers, just saying. Of course, I could collide with Lennard, like you can see on the photo, then somebody is injured, that is this crux, I really don’t want to judge that someone gets injured on the field, can also happen in a normal case, but that is quite a big responsibility.”

(line 44ff)

In this situation Norbert was self-reflective and was apparently conscious of the reasons for feeling exhausted. For him, it was a challenge to be engaged in unfamiliar situations, and he felt constricted because he could not act as he usually did. He remembered that he had to be very careful and was afraid of being injured, although there was no collision shown in the picture. This reveals he had not completely assimilated the new rules. Due to the well-defined zones on the playing field, the risk of collision is fairly small. An additional reason for his perceived uncertainty is embarrassment, a phenomenon that is found in a statement of Aron.

“Mmmh, because I never sat in one and I think I would not have the strength to move the wheelchair and I would need the time and when everyone is watching me, then I don’t know, I am under pressure and then maybe a lot of people will laugh.”

(line 109f)

Aron thinks that he lacks the skills to handle a wheelchair. He anticipated an unpleasant situation for himself and negative comments of his teammates. To avoid this situation, he did not try to play in a wheelchair. This reveals that Aron does not feel secure in playing handball in a wheelchair. These statements of perceived uncertainties are confirmed in the lower success rate of passes in comparison to the regular game (Table 1). It can be assumed that the lower success rate is directly connected to the feeling of uncertainty. However, the direction of the correlation remains unknown. That is to say, whether uncertainty leads to more mistakes or the higher number of mistakes leads to a feeling of uncertainty cannot be determined with the present data. The field notes suggest that problems with the rules and in the handling of the wheelchair had a negative effect on the number of shots on goal, which is a fundamental aspect of the game. The average values for the perception of skill (quantitative approach A) take a similar direction (Table 2). The values are relatively low, which shows a more negative perception of skill by participants without disabilities. A further description of the feeling of embarrassment was given in the interview with Björn:

“When we had the day with the wheelchairs, I sat in one and said to myself: ‘No, not for me.’ I don’t want to [do this] again, because I am happy, I am healthy, and I can walk.”

(line 71f)

It is obvious that Björn does not consider a wheelchair as sports equipment but as another degree of disability that he wants to avoid.

#### 7.1.3. Category: Positive New Experiences in the Wheelchair

Nevertheless, many athletes also had a positive report of playing in a wheelchair. They proudly described their experience of new movements and playing options. Furthermore, they gained an insight into the perspective of wheelchair users, as Max points out.

“Yes, I actually thought it was really good, because you see how it is from the wheelchair user’s point of view, because we are also not allowed to wheel around with their normal wheelchairs, instead have to use special wheelchairs. And then I thought it was good that we also tried it out … Yes, I had a lot of fun.”

(line 31ff)

Max noticed a difference between the wheelchairs used for exercise and the ones used for everyday mobility. Although he does not go into details about reasons, he explained that the new game was fun and that he enjoyed trying it out as well as getting a better insight into the perspective of wheelchair users. These positive experiences are not corroborated in the questionnaire data on athletes without disabilities. The categories special movement experiences and level of enjoyment of the physical activity only had medium values (Table 2).

Multiple statements by coaches underline the specific challenges concerning the movement of the wheelchair, which are included in the field notes under the code special movement experience. This applies to both players with and without disabilities:

“… unusual to be in a wheelchair, both players with and without disabilities are twisting with the wheelchair and show little control’ (line 44). Particularly with regard to the passing technique, the players tried different options: ‘the question arises whether a direct or indirect pass to a wheelchair player is best.”

(line 55)

It can be assumed that the new physical requirements are at least partly challenging and, therefore, perceived more positively (medium values on the scale for enjoyment of the physical activity, Table 2).

#### 7.1.4. Category: Wheelchair as a Benefit

Several athletes referred to the new possibilities of movement in a wheelchair. The modifications to the game made it slower. Athletes who found it difficult to keep up with the pace in a regular game benefited from this development. According to Ahmet, the wheelchair can assist players with disabilities.

“Well, yes, well, I thought it was good, with, with a wheelchair. Because, yes, well, I can walk, not so, good now. Well, I walk slowly, because of my foot. And with the wheelchair, yes, well, then I came forward fast, to the goal now. And that was good.”

(line 17f)

For Ahmet, a wheelchair allows him to enjoy participation. He can apparently move faster and, therefore, reach the goal faster, which Ahmet experiences as a moment of success. The results of video analysis confirm that the flow of the game decreased due to fewer passes (≈49%) than in the regular game (Table 1). However, Ahmet’s description reveals that the slowdown of the game can have a positive effect on inclusive participation in the game. The data show that the ratio of on time to off time is similar to that of a regular game (Table 1). More than 70% of the match time is on time (when scoring is possible). The number of game-related actions per minute (density) is also similar to that in the regular game. The data confirm that the modified game is indeed less dynamic than the regular game. However, in relation to on time and density of actions, player experience is similar and can, therefore, be assessed positively.

On the contrary, for players without disabilities, this change goes along with low values on the perceived skill scale (Table 2). This ambiguity is also noticeable in the following interpretation of the general categories.

### 7.2. Interpretation of the General Categories

With a view to the results in general, two perspectives emerge. They are grouped into the following general categories: wheelchair as disability and wheelchair as opportunity (Figure 2). Several statements by athletes can be clearly assigned to one of these general categories, while others advance both views and still others are ambiguous and cannot be clearly assigned to either of the general categories.

#### 7.2.1. General Category: Team Handball with Wheelchair as Disability

Most of the statements from the categories modified game and uncertain playing in the wheelchair can be assigned to this general category. Players with disabilities expressed their aversion to inclusive handball, mentioning reasons such as the perceived difference of the idea of the game, excessive tactical and technical demands, and loss of fun and enjoyment. In consequence, some players with disabilities did not want to play the modified game again.

Furthermore, players without disabilities perceived their own skills more negatively (Table 2). In general, the field notes confirm this aspect, but they also revealed learning effects. Additionally, the skills of the players with disabilities in wheelchairs were negatively perceived:

“The player without a disability often tried to pass the ball to a player with a disability in a wheelchair; several times he couldn’t catch the ball until the ball was rolling to the opponent.”

(line 8)

Such difficulties could be based on a lack of experience and remedied over time. The results of the video analysis do not confirm this finding because the success rate is quite similar between runners and those using wheelchairs (Table 1). Furthermore, positively perceived situations are observed according to perceived skills in passing:

“The other team is playing well from position to position, mostly everyone was in possession of the ball before a shot on the goal or an interception follows.”

(line 38)

Even according to the coding of enjoyment of the physical activity, the results draw a heterogeneous picture. Negatively associated observations “Wheelchair users are standing still and have no idea what to do. Boring!” stand in contrast to positively perceived emotions “But even excitement and joy was shown through laughing and clapping when a player with a disability was trying to play in a wheelchair and.” (line 75). 

This ambiguous picture is supported by the values of the enjoyment scale of the players without disabilities: the medium values show a high standard deviation.

Several times the field notes on social interaction and support show the goal-oriented interaction in the game. Negative interaction and missing support were always associated with non-disabled players:

“In this game, the behaviour of a player without a [visible] disability was very conspicuous. He played very egoistically, meaning as soon as he was in possession, he didn’t pass the ball anymore. He shouted at his teammates and also complained about decisions by the referee. Once he verbally demanded a pass to himself even though his teammate was unmarked.”

(line 81)

Intriguingly, in the questionnaires, the players without disabilities reported the highest values in the category social interaction and support. Though, the high standard deviation shows a heterogeneous rating (Table 2). This ambiguity is also visible in the results of the IOS. A part of the participants perceived themselves as members of the group; another part did not feel integrated (Table 2).

#### 7.2.2. General Category: Team Handball with Wheelchair as Opportunity

This general category sums up various statements from the categories new positive experience in the wheelchair and wheelchair as a benefit. Athletes described experiences and new opportunities that are made possible by playing in a wheelchair and playing together with both runners and those using wheelchairs. It becomes clear that many athletes recognize and accept the challenge facing wheelchair users. The players differ in their skills, needs, and perspectives on the sport. Dealing effectively with heterogeneity is crucial in inclusive teams if they are to meet the needs of all participants and to be able to adequately challenge and develop their individual abilities. Many athletes were aware of this and suggested working together to find solutions to improve participation by those using wheelchairs. They were also willing to play inclusive handball again.

Concerning group thinking, the coaches’ statements were ambivalent; the majority of positive statements referred to joint action as a group:

“Playing together worked well. Each player, whether with or without a disability, or player or wheelchair user, participated.”

(line 152)

It was also noticeable that the focus is on joint action. The data on the game experience did not allow a conclusion to be drawn about group thinking but described the actions in the group. The density of action for all players (per minute) showed that no player was completely excluded from the game, and thereby, everyone contributed to realizing the idea of the game. All players were actually involved in every minute of play (Table 1). The above-average values for the categories social interaction and support of players with disabilities (Table 2) also show that inclusive handball at this level can be regarded as positive.

In the category social interaction and support, the coaches expressed the particularly positive effects of inclusive handball on communication:

“In this game, the communication between the players was particularly noticeable.”

(line 90)

This is consistent with the quantitative data of the non-disabled players (SIS, Table 2).

## 8. Discussion

The results show different possibilities and limitations of the game form “inclusive handball” from the perspective of the athletes with intellectual disabilities. On the one hand, the changed game was described as rather negative (in contrast to the familiar handball game without wheelchair zones), and the players reported insecurities with the wheelchairs and the changed situation. It should be noted here that people with intellectual disabilities are often particularly insecure in situations that are new to them. It is, therefore, quite possible that the players with intellectual disabilities need more experience with the game in order to overcome their uncertainty. In previous studies on Freiwurf, coaches report exactly this effect [9]. However, when designing such an offer, it must be weighed very clearly what can be expected of the players with intellectual disabilities.

On the other hand, the players reported new positive experiences that they would not have had without playing in a wheelchair. In addition, many players saw the possibility that the wheelchair compensates for something and offers equal opportunities for everyone in the game. The question here is whether the game form “inclusive handball” offers a possibility to provide a game for all athletes (regardless of disability) in which everyone has the same conditions. One could assume this, as the runners can decide whether or not to play (temporarily) in a wheelchair. One of the biggest advantages is that wheelchair users can generally play along, which is not possible in normal team handball. This shows strong advantages of the game form, which are also supported by the evaluation. This choice is an outstanding component of a club sport offer in the sense of the UN Convention [8].

With respect to the interaction of people with and without disabilities, ambiguous phenomena became visible. Runners with intellectual disabilities reported that learning about the outgroup of wheelchair users takes place primarily through trying out the game in a wheelchair. The valourization of one’s ingroup (athletes with intellectual disabilities) in this case study also differed from previous findings of studies on Freiwurf [5] and also studies on unified sports [6,7]. Classifications of disabled or non-disabled were not found in the case study data. Instead, a distinction was made between players in wheelchairs and runners. Furthermore, some athletes with intellectual disabilities experienced themselves as more skilled in the position of runner than as wheelchair athletes. It is also clear that athletic ability is important here for the evaluation of the group.

The favourable conditions for contact between people with and without disabilities defined by Cloerkes [16] are present in inclusive handball. Similar to other inclusive handball games [5,7,9], the intensity of contact seems to be high. One possible difference seems to be that players without disabilities perceive less social reward, as the distinction between players with and without disabilities is partly replaced by the distinction between wheelchair users versus runners, with players with disabilities sometimes taking on a helping role in the game.

The data beyond the interviews offer further insights into this. Thus, the blending of data can be seen as effective. One example is that the game dynamics tends to be evaluated negatively in comparison to that of regular team handball games, but the reduced pace of the game also seems to have positive aspects. All four methods of data collection show that communication and social interactions as well as support were evaluated positively.

The participants’ criticism expressed in the interviews, which was also corroborated by the further data, can be used for further development of such inclusive forms of play. The main points of criticism from the players were the lower number of passes, the higher error rate, and difficulties with non-disabled athletes in wheelchairs. The hypothesis is that with habituation to the rules and technical and tactical development through practice (improvements were already observed during the intervention), the game dynamics will adjust to a higher level. If this is the case, the physical and cognitive advantages of non-disabled players competing in a wheelchair will be compensated by the use of a wheelchair. It might then be possible to remove the mental division of inclusive handball players into those with and those without disabilities, as the “new shared disability” helps to create a game with equal opportunities. This development would be an important element in promoting true inclusion in team sports such as team handball.

### 8.1. Limitations

The generalizability of the study is limited as it is a case study in the context of Freiwurf Hamburg. Thus, the results should be written with care regarding other samples or groups and are just an expression of the behaviours and thinking in the specific context of the Freiwurf community. Further, the number of players without disabilities who participated in the questionnaire surveys was rather small, and therefore the descriptive results are as well limited to this specific group.

### 8.2. Conclusions

The case study shows that inclusive handball is a way to let male and female players with and without disabilities, in wheelchairs or as runners, play with and against each other in a handball competition. This form of the game has been specially developed for the special situation of Freiwurf and has addressed the problem of wheelchair users not being part of the game. From the solution of the problem, the form of the game “inclusive handball”, arose at the same time a form of the game that also pushes the idea that really all players act with equal chances. Here, we can see that diversity and the will for real inclusion can lead to useful solutions and is enriching.

It is important to be courageous and creative and also to develop and try out specific ideas for inclusive teams. The form of the game presented here may be transferable to other teams. It is very innovative, especially because wheelchair users and runners can play together for the first time.

## Figures and Tables

**Table 1:** Frequency and percentage (per match) of the parameters of the game experience of runners (R) and wheelchair users (WU) playing inclusive handball as well as the regular Freiwurf game.

	Inclusive Handball	Regular Freiwurf Game
On time (%)	76.05 (SD = 4.36)	71.78 (SD = 3.61)
Successful passes (*n*)	R 54.75 (SD = 10.37) WU 28.50 (SD = 8.10)	168 (SD = 19.31)
Successful passes (%)	R 81.83 (SD = 2.08) WU 83.85 (SD = 6.52)	97.33 (SD = 1.35)
Actions/player/minute (*n*)	1.28 (SD = 0.65)	1.31 (SD = 0.19)

**Table 2:** Means (M) and standard deviation (SD) of the scales of group thinking and sports enjoyment; *n* = 10; 4 male, 6 female; age M = 35.6; SD = 13.31; experience in team handball in years M = 12.30, SD = 14.41.

	Group-Thinking IOS Scale	Sports Enjoyment			
		PS	SIS	SME	EPA
M	3.50	2.43	4.03	3.18	3.43
SD	2.27	1.33	1.43	1.19	1.32

Note. PS = perceived skill, SIS = social interaction and support, SME = special movement experience, EPA = enjoyment of the physical activity.

## Data Availability

Detailed data can be obtained from the authors.
